# Log-logistic distribution for survival data analysis using MCMC

**DOI:** 10.1186/s40064-016-3476-7

**Published:** 2016-10-12

**Authors:** Ali A. Al-Shomrani, A. I. Shawky, Osama H. Arif, Muhammad Aslam

**Affiliations:** Department of Statistics, Faculty of Science, King Abdulaziz University, P.O. Box 80203, Jeddah, 21589 Saudi Arabia

**Keywords:** Log-logistic, Posterior, Non-informative, Module, OpenBUGS, Uniform priors

## Abstract

This paper focuses on the application of Markov Chain Monte Carlo (MCMC) technique for estimating the parameters of log-logistic (LL) distribution which is dependent on a complete sample. To find Bayesian estimates for the parameters of the LL model OpenBUGS—established software for Bayesian analysis based on MCMC technique, is employed. It is presumed that samples for independent non informative set of priors for estimating LL parameters are drawn from posterior density function. A proposed module was developed and incorporated in OpenBUGS to estimate the Bayes estimators of the LL distribution. It is shown that statistically consistent parameter estimates and their respective credible intervals can be constructed through the use of OpenBUGS. Finally comparison of maximum likelihood estimate and Bayes estimates is carried out using three plots. Additively through this research it is established that computationally MCMC technique can be effortlessly put into practice. Elaborate procedure for applying MCMC, to estimate parameters of LL model, is demonstrated by making use of real survival data relating to bladder cancer patients.

## Background

The log-logistic (LL) distribution (branded as the Fisk distribution in economics) possesses a rather supple functional form. The LL distribution is among the class of survival time parametric models where the hazard rate initially increases and then decreases and at times can be hump-shaped. The LL distribution can be used as a suitable substitute for Weibull distribution. It is in fact a mixture of Gompertz distribution and Gamma distribution with the value of the mean and the variance coincide—equal to one. The LL distribution as a life testing model has its own standing; it is an increasing failure rate (IFR) model and also is viewed as a weighted exponential distribution.

Scrolling through the literature on the subject distribution we see that Bain (1974) modeled LL distribution by a transformation of a well-known logistic variate. The properties of LL distribution have been deliberated upon by Ragab and Green (1984) who also worked on the order statistics for the said distribution. Kantam et al. (2001) proposed acceptance sampling plan using the LL distribution. Kantam et al. (2006) designed economic acceptance sampling plan using the LL distribution. Kantam and Rao (2002) derived the modified maximum likelihood estimation (MLE) of this distribution. Rosaiah et al. (2007) derived confidence intervals using the LL model-approximation to ML method. The properties, estimation and testing of linear failure rate using exponential and half-logistic distribution has been discussed thoroughly by Rao et al. (2013). Rosaiah et al. (2014) studied the exponential-LL distribution additive failure rate.

The current research intends to use LL distribution for modeling the survival data and to obtain MLE utilizing associated probability intervals of the Bayes estimates. It has been noticed that the Bayesian estimates may not be computed plainly under the assumption of independent uniform priors for the parameters. The authors will work under the assumption that both parameters—shape and scale, of the LL model are unknown.

The authors will develop the algorithm to generate Markov Chain Monte Carlo (MCMC) samples based on the generated posterior samples from the posterior density function using Gibbs sampling technique by employing the OpenBUGS software. Bayesian estimates of parameters along with highest posterior density (HPD) credible intervals will be constructed. Moreover, estimation of the reliability function will also be looked into. Entire statistical computations and functions for LL will be done using R statistical software see Lyu (1996), Srivastava and Kumar (2011a, b, c) and Kumar et al. (2012, 2013). Real life data will be considered, in order to illustrate how the proposed technique can be effortlessly applied in an orderly manner in real life situations.

Remainder of the paper contains six sections: “Model analysis”, “Maximum likelihood estimation, (MLE) and information matrix”, “Model validation”, “Bayesian estimation using Markov Chain Monte Carlo (MCMC) method”; “Comparison of MLE estimates and Bayes estimates” and “Conclusion”.

## Model analysis

### Probability density function (pdf)

If a r.v X has a LL distribution having shape parameter $\alpha > 0$ plus scale parameter $\lambda > 0,$ denoted by *X* ~ LL $\left( {\alpha , \lambda } \right).$ The pdf of the LL distribution is of the form:

1 $$f\left( {x; \alpha , \lambda } \right) = \frac{{\left( {\alpha /\lambda } \right)\left( {x /\lambda } \right)^{\alpha - 1} }}{{\left( {1 + \left( {x /\lambda } \right)^{\alpha } } \right)^{2} }},\quad \alpha > 0,\quad \lambda > 0, \quad x \ge 0.$$

### Cumulative density function (CDF)

The CDF of the LL model with two parameters takes the form;

2 $$F\left( {x; \alpha , \lambda } \right) = 1 /\left( {1 + \left( {x /\lambda } \right)^{ - \alpha } } \right) ,\quad \alpha > 0,\quad \lambda > 0,\quad x \ge 0.$$

### The reliability function

The reliability (survival) function of LL model takes the form;

3 $$R\left( {x; \alpha , \lambda } \right) = \left( {x /\lambda } \right)^{ - \alpha } /\left( {1 + \left( {x /\lambda } \right)^{ - \alpha } } \right), \quad \alpha > 0,\quad \lambda > 0, \quad x \ge 0.$$

### The Hazard function

The hazard rate function of LL model is

4 $$h\left( {x; \alpha , \lambda } \right) = \frac{\alpha }{{x\left[ {1 + \left( {x/\lambda } \right)^{ - \alpha } } \right]}}.$$

### The cumulative hazard function H(x)

H(x) of LL model takes the form;

5 $$H\left( x \right) = - logR\left( x \right) = \int_{0}^{x} {h\left( t \right)dt.}$$

### The failure rate average (FRA) and conditional survival function (CSF)

Two additionally useful reliability functions are FRA and CSF (Rausand and Hoyland 2004). The FRA of X is;

6 $$FRA\left( x \right) = \frac{H\left( x \right)}{x} = \frac{{\mathop \int \nolimits_{0}^{x} h\left( x \right)dx}}{x},\quad x > 0,$$

where, *H*(*x*) is the cumulative hazard function.

An analysis of FRA (*x*) on *x* enables us to find increasing failure rate average (IFRA) and decreasing failure rate average (DFRA).

The survival function (SF) and the conditional survival of X are defined respectively, by (Rausand and Hoyland 2004).

$$R\left( x \right) = 1 - F\left( x \right),$$

and

7 $$P(X > x + t\left| {X > t) = } \right.R(x\left| {t) = } \right.\frac{{R\left( {x + t} \right)}}{R\left( x \right)}, \quad t > 0, \quad x > 0,\quad R\left( \cdot \right) > 0,$$

where F(*x*) is the CDF of *x* analogous to H(*x*) in FRA(x), the distribution of *x* belongs to the new better than used (NBU), exponential, or new worse than used (NWU) classes, when R(*x*|t) < R(*x*), R(*x*|t) = R(*x*), or R(*x*|t) > R(*x*), respectively, see Rausand and Hoyland (2004) and Lai and Xie (2006).

### The quantile function

The quantile function of LL model is;

8 $$x_{q} = \lambda \left( {q^{ - 1} - 1} \right)^{ - 1/\alpha } ,\quad 0 < q < 1.$$

### The random deviate generation functions

Let U be a random variable which follows uniform distribution (0,1) with CDF, F(·) for which inverse exists. Then any sample drawn from F^−1^(u) is considered to be drawn from F(·). So, the random deviate can be generated from LL ($\alpha , \;\lambda$) using

9 $$x = \lambda \left( {u^{ - 1} - 1} \right)^{ - 1/\alpha } ,\quad 0 < u < 1.$$

where; u follows U(0,1) distribution.

## Maximum likelihood estimation (MLE) and information matrix

MLEs of the two-parameter LL model plus their large sample properties in order to find approximate confidence intervals based on MLEs are discussed in this section.

Suppose $x = \left( {x_{1} , x_{2} , \ldots , x_{n} } \right)$ be an observed sample of size *n* from LL model, in that case the log-likelihood function L ($\alpha ,\, \lambda$, $\lambda$) is given as (Singh and Guo 1995).

10 $$\begin{aligned} \ell & = {\text{log L}} = {\text{n}}\,\log\,{\upalpha } - {\text{n}}\,\log\,{\uplambda } + \left( {{\upalpha } - 1} \right)\mathop \sum \limits_{{{\text{i}} = 1}}^{\text{n}} \log\, {\text{x}}_{\text{i}} - {\text{n}}\left( {{\upalpha } - 1} \right){ \log \uplambda } \\ & \quad - 2\mathop \sum \limits_{{{\text{i}} = 1}}^{\text{n}} { \log }\left[ {1 + \left( {\frac{{{\text{x}}_{\text{i}} }}{\uplambda }} \right)^{\upalpha } } \right]. \\ \end{aligned}$$

To obtain the MLEs of the two parameters α and λ, maximize (10) directly with respect to *α* and *λ* or, otherwise may be solved using Newton–Raphson method.

11 $$\frac{\partial \ell }{\partial \alpha } = \frac{n}{\alpha } + \mathop \sum \limits_{{{\text{i}} = 1}}^{\text{n}} \log {\text{x}}_{\text{i}} - {\text{n}}\,\log\,{\uplambda } - 2\mathop \sum \limits_{{{\text{i}} = 1}}^{\text{n}} \frac{{\left( {\frac{{{\text{x}}_{\text{i}} }}{\uplambda }} \right)^{\upalpha } \log \left( {\frac{{{\text{x}}_{\text{i}} }}{\uplambda }} \right)}}{{1 + \left( {\frac{{{\text{x}}_{\text{i}} }}{\uplambda }} \right)^{\upalpha } }} = 0,$$

12 $$\frac{\partial \ell }{\partial \lambda } = - \frac{n}{\lambda } - \frac{{n\left( {\alpha - 1} \right)}}{\lambda } + \frac{2\alpha }{\lambda }\mathop \sum \limits_{i = 1}^{n} \frac{{\left( {\frac{{{\text{x}}_{\text{i}} }}{\uplambda }} \right)^{\upalpha } }}{{1 + \left( {\frac{{{\text{x}}_{\text{i}} }}{\uplambda }} \right)^{\upalpha } }} = 0.$$

### Information matrix and asymptotic confidence intervals

Let us denote, parameter vector by $\underline{\delta } = \left( {\alpha , \lambda } \right)$ and the corresponding MLE of $\underline{\delta }$ as $\underline{{\hat{\delta }}} = \left( {\hat{\alpha }, \hat{\lambda }} \right)$, then the asymptotic normality results can be written in the following form

13 $$(\underline{{{{\hat{\updelta }}}}} - \underline{\updelta } ) \to {\text{N}}_{2} \left( {0,({\text{I}}\left( {\underline{\updelta } } \right))^{ - 1} } \right),$$

where I($\underline{\delta }$) is the Fisher’s information matrix (FIM) is obtained by

14 $${\text{I}}\left( {\underline{\updelta } } \right) = - \left[ {\begin{array}{*{20}c} {E\left( {\frac{{\partial^{2} \ell }}{{\partial \alpha^{2} }}} \right)} & {E\left( {\frac{{\partial^{2} \ell }}{\partial \alpha \partial \lambda }} \right)} \\ {E\left( {\frac{{\partial^{2} \ell }}{\partial \alpha \partial \lambda }} \right)} & {E\left( {\frac{{\partial^{2} \ell }}{{\partial \lambda^{2} }}} \right)} \\ \end{array} } \right].$$

Since $\underline{\updelta }$ is unknown therefore it is useless to have an asymptotic variance $({\text{I}}\left( {\underline{\updelta } } \right))^{ - 1}$ for the MLEs. So, the asymptotic variance can be approximated by “installing in” the estimated values of the parameters, see Lawless (2003). Modus operandi under such a situation is to make use of the observed FIM O($\underline{{\hat{\delta }}}$) (as an estimate of I $(\underline{\updelta } )$) and it is given by

15 $${\text{O}}\left( {\underline{{\hat{\delta }}} } \right) = - \left[ {\begin{array}{*{20}c} {\frac{{\partial^{2} \ell }}{{\partial \alpha^{2} }}} & {\frac{{\partial^{2} \ell }}{\partial \alpha \partial \lambda }} \\ {\frac{{\partial^{2} \ell }}{\partial \alpha \partial \lambda }} & {\frac{{\partial^{2} \ell }}{{\partial \lambda^{2} }}} \\ \end{array} } \right]_{{\left| {\left( {\hat{\alpha }, \hat{\lambda }} \right)} \right.}} = - H\left( {\underline{\updelta } } \right)_{{\left| {\underline{{\hat{\delta }}} } \right.}} ,$$

where, H is known as Hessian matrix.

Here the Newton–Raphson algorithm comes handy which in fact maximizes the likelihood, produces the observed information matrix and consequently the variance–covariance matrix is given as;

16 $$\left( { - H\left( {\underline{\updelta } } \right)_{\vert{{\underline{{\hat{\delta }}} }} }} \right)^{ - 1} = \left( {\begin{array}{*{20}c} {Var\left( {\hat{\alpha }} \right)} & {Cov\left( {\hat{\alpha }, \hat{\lambda }} \right) } \\ {Cov\left( {\hat{\alpha }, \hat{\lambda }} \right)} & {Var\left( {\hat{\lambda }} \right)} \\ \end{array} } \right).$$

By virtue of asymptotic normality of MLEs, approximate 100(1 − $\gamma$)% confidence intervals for $\alpha$ and $\lambda$ can be constructed as

$$\hat{\alpha } \pm Z_{\gamma /2} \sqrt {Var\left( {\hat{\alpha }} \right)} \;{\text{and}}\;\hat{\lambda } \pm Z_{\gamma /2} \sqrt {Var\left( {\hat{\lambda }} \right)} ,$$

where $Z_{\gamma /2}$ is the upper percentile of standard normal variate.

### Computation of maximum likelihood estimation

In order to have insight into the ML estimation a data has been adapted from Lee and Wang (2003). The sample data consists of 128 patients having bladder cancer and the values shown are the monthly remission times.

0.08, 2.09, 3.48, 4.87, 6.94, 8.66, 13.11, 23.63, 0.20, 2.23, 3.52, 4.98, 6.97, 9.02, 13.29, 0.40, 2.26, 3.57, 5.06, 7.09, 9.22, 13.80, 25.74, 0.50, 2.46, 3.64, 5.09, 7.26, 9.47, 14.24, 25.82, 0.51, 2.54, 3.70, 5.17, 7.28, 9.74, 14.76, 26.31, 0.81, 2.62, 3.82, 5.32, 7.32, 10.06, 14.77, 32.15, 2.64, 3.88, 5.32, 7.39, 10.34, 14.83, 34.26, 0.90, 2.69, 4.18, 5.34, 7.59, 0.66, 15.96, 36.66, 1.05, 2.69, 4.23, 5.41, 7.62, 10.75, 16.62, 43.01, 1.19, 2.75, 4.26, 5.41, 7.63, 17.12, 46.12, 1.26, 2.83, 4.33, 5.49, 7.66, 11.25, 17.14, 79.05, 1.35, 2.87, 5.62, 7.87, 11.64, 17.36, 1.40, 3.02, 4.34, 5.71, 7.93, 11.79, 18.10, 1.46, 4.40, 5.85, 8.26, 11.98, 19.13, 1.76, 3.25, 4.50, 6.25, 8.37, 12.02, 2.02, 3.31, 4.51, 6.54, 8.53, 12.03, 20.28, 2.02, 3.36, 6.76, 12.07, 21.73, 2.07, 3.36, 6.93, 8.65, 12.63, 22.69.

The values calculated for the mean, variance and the coefficient of skewness are $9.36562, 110.425 \;{\text{and}}\; 3.32567,$ respectively. The measure of skewness indicates that data are positively skewed whereas the coefficient of skewness is the unbiased estimator for the population skewness obtained by $= \frac{{\sqrt {n\left( {n - 1} \right)} }}{n - 2}\cdot\frac{{\frac{1}{n}\mathop \sum \nolimits_{i = 1}^{n} \left( {x_{i} - \bar{x}} \right)^{3} }}{{\left( {\frac{1}{n}\mathop \sum \nolimits_{i = 1}^{n} \left( {x_{i} - \bar{x}} \right)^{2} } \right)^{3/2} }}.$

The data is fitted using the LLmodel. *Optim* () function in R with Newton–Raphson options was used as an iterative process for maximizing the log-likelihood function given in (10). The values of the estimates thus obtained are $\hat{\alpha } =$ 1.725158, $\hat{\lambda } =$  6.089820 and the related log-likelihood value = −411.4575 is obtained by using *maxLik* package available in R. An estimate of variance–covariance matrix, using (15) and (16), is given as

$$\left( {\begin{array}{*{20}c} {Var\left( {\hat{\alpha }} \right)} & {Cov\left( {\hat{\alpha }, \hat{\lambda }} \right) } \\ {Cov\left( {\hat{\alpha }, \hat{\lambda }} \right)} & {Var\left( {\hat{\lambda }} \right)} \\ \end{array} } \right) = \left( {\begin{array}{*{20}c} {61.127411} & { - 0.331139 } \\ { - 0.331139 } & {3.451350} \\ \end{array} } \right).$$

Equation (16) was used to construct the 95 % confidence intervals for the parameters of LL model using on MLE’s. Table 1 displays the MLE’s along with their standard errors and approximate 95 % confidence intervals for $\alpha$ and $\lambda$.

**Table 1:** Maximum likelihood estimates, standard errors and 95 % confidence intervals

Parameter	MLE	SE	95 % confidence interval
Alpha	1.725158	0.1279366	(1.474407, 1.975909)
Lambda	6.089820	0.5384165	(5.034543, 7.145097)

## Model validation

Srivastava and Kumar (2011c) suggest than in order to assess the goodness of fit of the proposed LL model, it is essential to work out the Kolmogorov–Smirnov (K–S) statistics between the empirical distribution function and the fitted LL model. The authors found the fit to be appropriate since the value of the K–S test i.e. D = 0.03207318 had the sig. value of 0.998 which is far greater than the predetermined level of 0.05. Therefore, it can be confidently asserted that the proposed LL model is appropriate to analyze the data set.

**Fig. 1:**
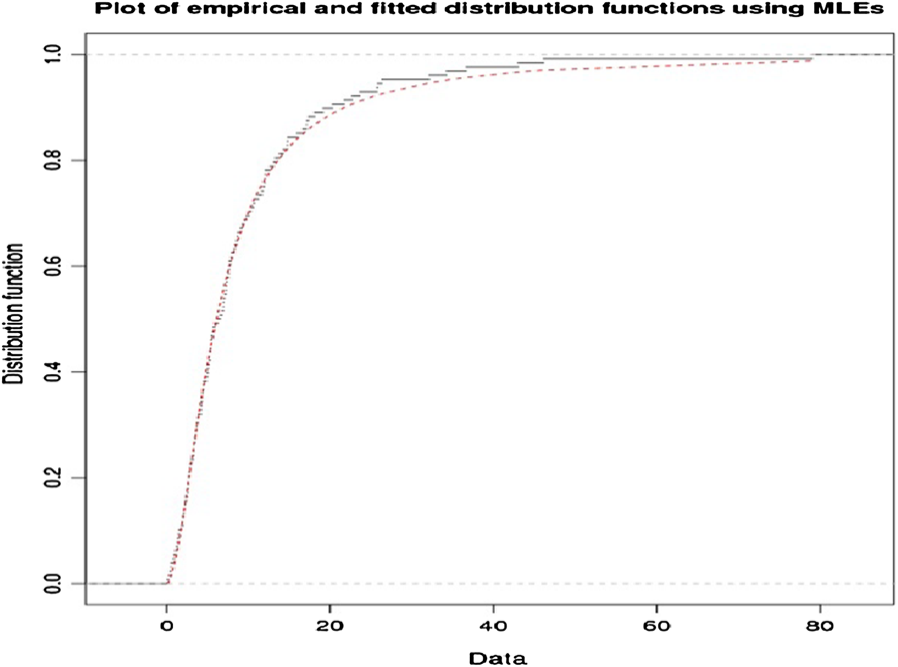
The graph of empirical and fitted distribution function

To further supplement our claim of goodness of fit, both the empirical and fitted distribution functions are displayed in Fig. 1. It is quite evident that there is a reasonable coincided match between the two distributions. Keeping in view the foregoing results, we feel confident in expressing that the estimated LL model gives a good fit.

For model validation quantile- quantile (Q–Q) and probability–probability (P–P) plots are most commonly used graphical methods to assess whether the fitted model is in agreement with the given data.

Suppose ${\hat{\text{F}}}\left( {\text{x}} \right)$ be an estimate of F(x) based on $x_{1} , x_{2} , \ldots , x_{n} .$ The scatter diagram of the points

$${\hat{\text{F}}}^{ - 1} \left( {{\text{p}}_{{1:{\text{n}}}} } \right)\;{\text{versus}}\; {\text{x}}_{{{\text{i}}:{\text{n}}}} ,\;{\text{i }} = \, 1,2, \ldots ,{\text{n}},\;{\text{will be a q}}\text{-}{\text{q plot}} .$$

**Fig. 2:**
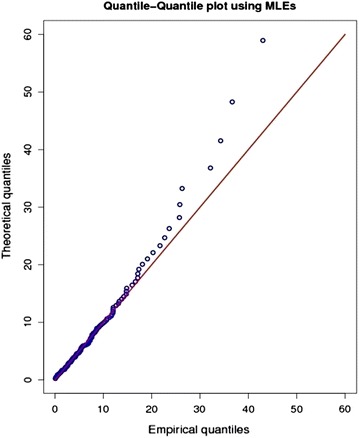
Quantile–quantile (Q–Q) plot using MLEs as estimate

The q–q plot shows the estimated versus the observed quantiles. If the model fits is good the of points on the q–q plot will roughly exhibit a 45° straight line. From Fig. 2 we see that approximately straight line pattern appears suggesting that the LL model offers a good fit.

**Fig. 3:**
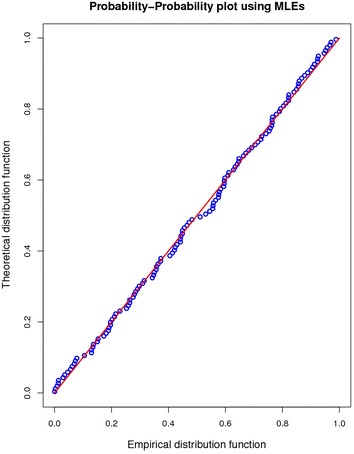
p–p plot using MLEs

Likewise the foregone claim is also supplemented by the p–p plot in Fig. 3. Suppose $x_{1} , x_{2} , \ldots , x_{n}$ be a sample from a given population with estimated cdf ${\hat{\text{F}}}\left( {\text{x}} \right)$. The scatter diagram of ${\hat{\text{F}}}\left( {{\text{x}}_{{1:{\text{n}}}} } \right)$ versus ${\text{p}}_{{{\text{i}}:{\text{n}}}} ,$ i = 1, 2, …, n, is known as a p–p plot. If the LL model fits is good, the points will be close to the 45° diagonal line, Srivastava and Kumar (2011b). Here again it is witnessed that maximum points in the p–p plot lie within the required range.

## Bayesian estimation using Markov Chain Monte Carlo (MCMC) method

Monte Carlo is repeated pseudo-random sampling generating technique. It makes use of algorithm to generate samples. Markov Chain on the other hand is a random process with a countable state-space with the Markov property. According to Chen et al. (2000) that Markov property means that the future state is dependent only on the present state and not on the past states. The combination of Markov chains and Monte Carlo techniques is commonly referred to as MCMC, see Robert and Casella (2004). Since the advent of the computed friendly software application of MCMC in Bayesian estimation has gained currency for the last one decade or so. Presently, for applied Bayesian inference, researchers usually work on OpenBUGS (Thomas 2010). It is a menu driven and, for existing probability models, contains a modular framework which is capable of being extended, if such a need arises, for constructing and evaluating Bayesian probability models (Lunn et al. 2000).

Since LL model is not a default probability model in OpenBUGS, therefore it warrants an integration of a module for parameter estimation of LL model. The Bayesian analysis of a probability model can be executed only for the default probability models in OpenBUGS. Of late, some probability models are integrated in OpenBUGS in order to ease the Bayesian analysis (Kumar et al. 2010). For more details about the OpenBUGS of some other models, the readers are referred to Kumar et al. (2012) and Srivastava and Kumar (2011a, b, c).

### Bayesian analysis under uniform priors

The proposed module is designed with a view to work out the Bayesian estimates for the LL model through MCMC technique. The primary purpose of the module is to generate, MCMC samples from posterior distribution for non-informative uniform priors. The norm is, that one is in know of the likely values of $\theta$ that occur over a finite range [a, b]. There are many other informative prior distributions such as gamma distribution, beta distribution and normal distribution. We are using non-informative uniform priors as we have no knowledge of the behaviour of parametric $\theta$. Because there is no idea about the value of parameter and we have only information about the lower and upper limits of $\theta$. With this situation at hand, a uniform distribution with a definite interval may be a reasonable guess of the prior distribution, and its PDF may be taken as;

$$\pi \left( \theta \right) = \left\{ {\begin{array}{*{20}l} {\frac{1}{b - a},} \hfill &\quad { 0 < a \le \theta \le b} \hfill \\ {0,} \hfill &\quad {otherwise.} \hfill \\ \end{array} } \right.$$

The authors initiated two parallel chains for sufficiently large number of iterations until the convergence is attained. For the current study the convergence was attained at 40,000 with a burn-in of 5000. Finally posterior sample of size 7000 is used by selecting a thinning interval of five i.e. every fifth outcome is stored. Thus, we have the posterior sample {$\alpha_{1i} ,\lambda_{1i}$ }, *i* = 1 … 7000 drawn from chain 1 and { $\alpha_{2i} ,\lambda_{2i}$ }, *i* = 1 … 7000 from chain 2. Chain 1 is earmarked for testing convergence. Whereas, chain 2 is earmarked for displaying visual summary. Both Chain 1 and Chain 2 shall be utilized for looking into the numerical summary.

### Convergence diagnostics

Simulation draws or chains were started at initial values for each parameter of priors. Due to dependency in successive draws, first draws were discarded as a burn-into obtain independent samples. Therefore, we need to be sure that the chains have converged in MCMC analysis in order to make inferences from the posterior distribution. This was checked by several diagnostic analyses as follows.

### History (trace) plot

**Fig. 4:**
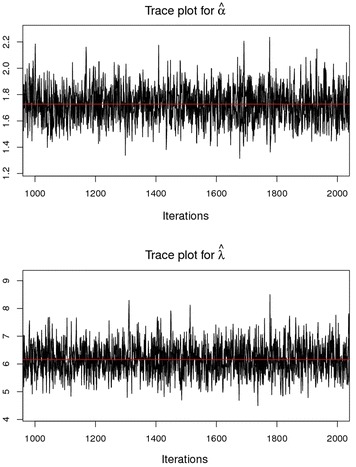
Sequential realization of the parameters *α* and *λ*

From the graphs in Fig. 4 we can safely conclude that the chains have converged as the plots exhibits no extended increasing or decreasing trends, rather it looks like a horizontal band.

### Autocorrelation plot

**Fig. 5:**
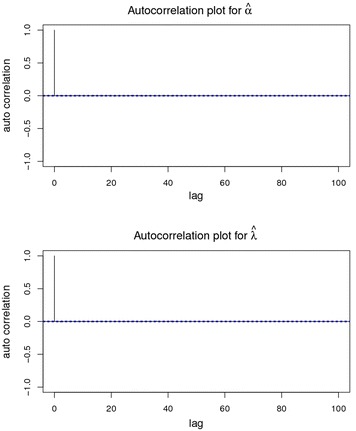
The autocorrelation plots for *α* and *λ*

Autocorrelation plots clearly indicate that the chains are not at all autocorrelated. The later part is better since samples from the posterior distribution contained more information about the parameters than the succeeding draws. Almost negligible correlation is witnessed from the graphs in Fig. 5. So the samples may be considered as independent samples from the target distribution, i.e. the posterior distribution.

### Visual summary through Kernel density estimates

**Fig. 6:**
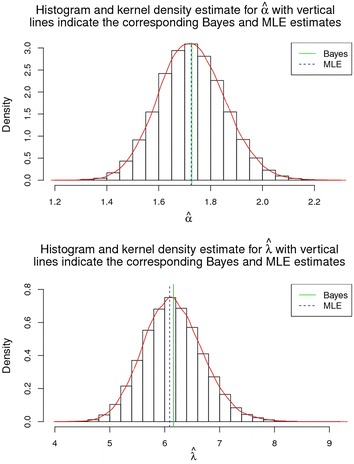
Histogram and kernel density estimate of *α* and *λ*

Samples drawn from chain 2 were earmarked for displaying visual summary for the LL model. Sufficient insight is provided by histograms regarding asymmetry, tail behaviour, multi-modal behaviour, and extreme values. Comparison of the histograms may also be carried out with other basic shapes related with standard diagnostic distributions. Histogram and kernel density estimate of $\alpha$ and $\lambda$ based on Chain 2 iterations, are displayed in Fig. 6 with vertical dotted line and thick line representing MLEs and Bayesian estimates respectively.

### Numerical summary

Chain 1 and chain 2 samples are used for looking into the numerical summary regarding LL model. Table 2 displays numerical values of ten quantities of interest, based on MCMC samples from posterior characteristics of LL model, under uniform priors. The numerical summary shown below is obtained from 7000 samples based on final posterior samples each for $\varvec{\alpha}$ and $\varvec{\lambda}.$

$$\left\{ {\alpha_{1i} ,\lambda_{1i} } \right\},\;i = \, 1, \ldots ,7000\;{\text{from}}\;{\text{chain}}\; 1 ,$$

and

$$\left\{ {\alpha_{2i} ,\lambda_{2i} } \right\},\;i = \, 1, \ldots ,7000\;{\text{from}}\;{\text{chain}}\; 2.$$

**Table 2:** Numerical summaries based on MCMC sample of posterior characteristics for LL model under uniform priors

Characteristics	Chain 1		Chain 2	
	*α*	*λ*	*α*	*λ*
Mean	1.728	6.160	1.728	6.159
SD	0.1271	0.5420	0.1275	0.5473
Naive SE	0.0006791	0.0028971	0.0006817	0.0029254
Time-series SE	0.000845	0.003621	0.0008483	0.0036895
Minimum	1.223	4.168	1.254	4.140
2.5th percentile (P_2.5_)	1.487	5.153	1.486	5.155
First quartile (Q_1_)	1.641	5.787	1.640	5.781
Median	1.725	6.142	1.725	6.136
Third quartile (Q_3_)	1.812	6.512	1.812	6.508
97.5th percentile (P_97.5_)	1.984	7.273	1.983	7.293
Maximum	2.278	9.171	2.284	8.826
95 % credible interval	1.487, 1.984	5.153, 7.273	1.486, 1.983	5.155, 7.293
95 % HPD credible interval	1.479, 1.796	5.093, 7.207	1.479, 1.976	5.142, 7.273

### Running mean (Ergodic mean) plot

**Fig. 7:**
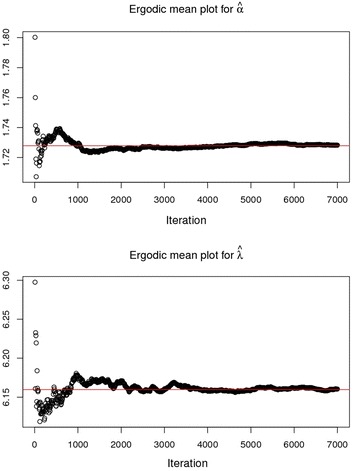
The Ergodic mean plots for α and *λ*

Convergence pattern of MCMC chain is observed by calculating running mean which is the overall mean of all samples up to and including a particular iteration. Time series graph of each parameter is generated from the chain commonly known as Ergodic mean plots. Figure 7 displays the Ergodic mean plots for the two parameters. It is quite clear from the Ergodic mean plot of alpha that the chain converges after 2000 iterations to the value of 1.728 and the Ergodic mean plot for lambda converges after 4000 iterations to the value of 6.16.

### Brooks–Gelman–Rubin plot

**Fig. 8:**
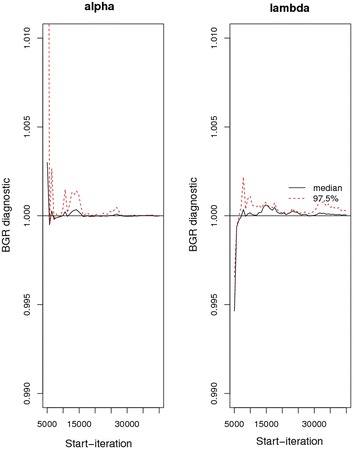
BGR plots for α and *λ*

The evidence of convergence from BGR plots displayed in Fig. 8 comes from the fact that the black line for both alpha and lambda converge to 1 and from the red line being steady (horizontal) across the breadth of the plot.

### Visual summary using box plots

**Fig. 9:**
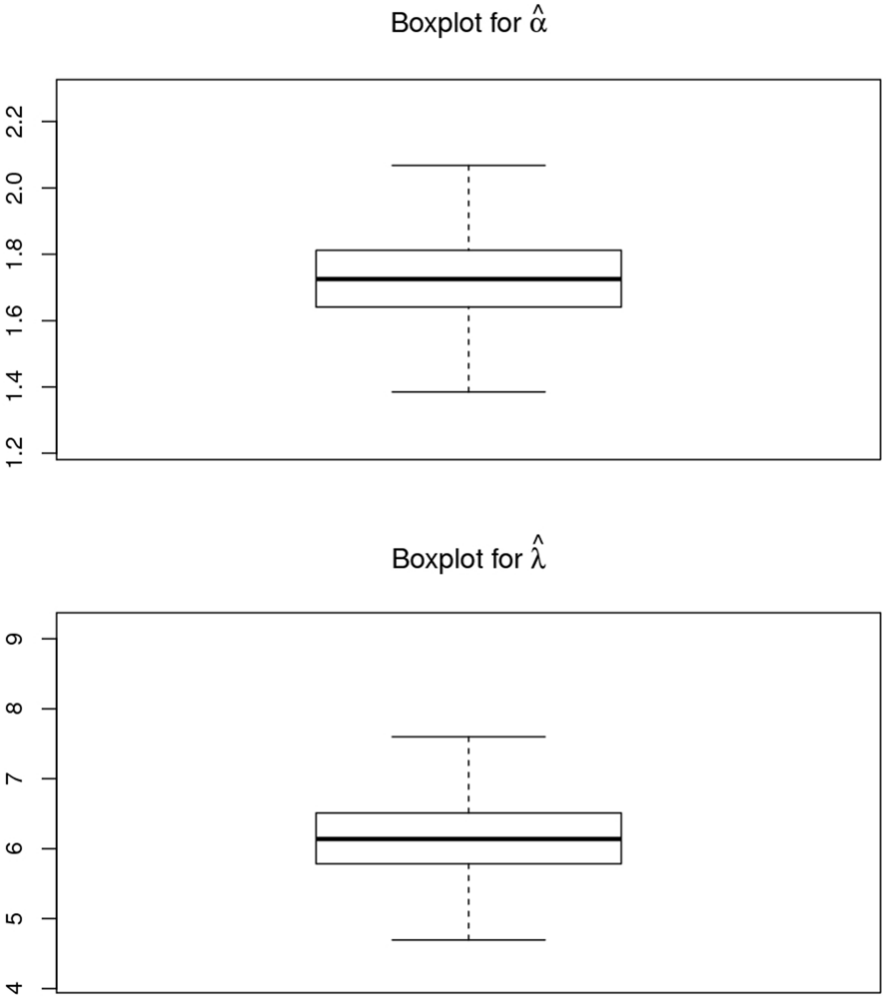
The boxplots for α and *λ*

The boxes in Fig. 9 symbolize inter-quartile ranges with the thick black line in the middle of the boxes represent means for alpha and lambda, the whiskers of each box depicts the middle 95 % of the distribution—the ends are in fact 2.5 percent and 97.5 percent quantiles.

## Comparison of MLE estimates and BAYES estimates

**Fig. 10:**
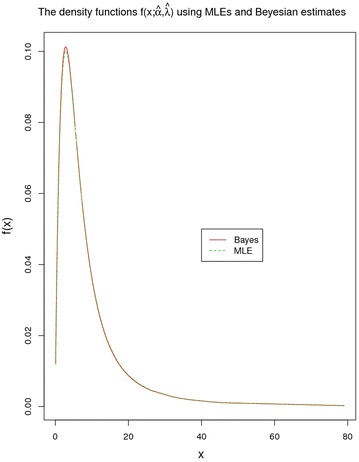
Density functions f(x,$\hat{\alpha }, \hat{\lambda }$)employing MLEs and Bayesian estimates

**Fig. 11:**
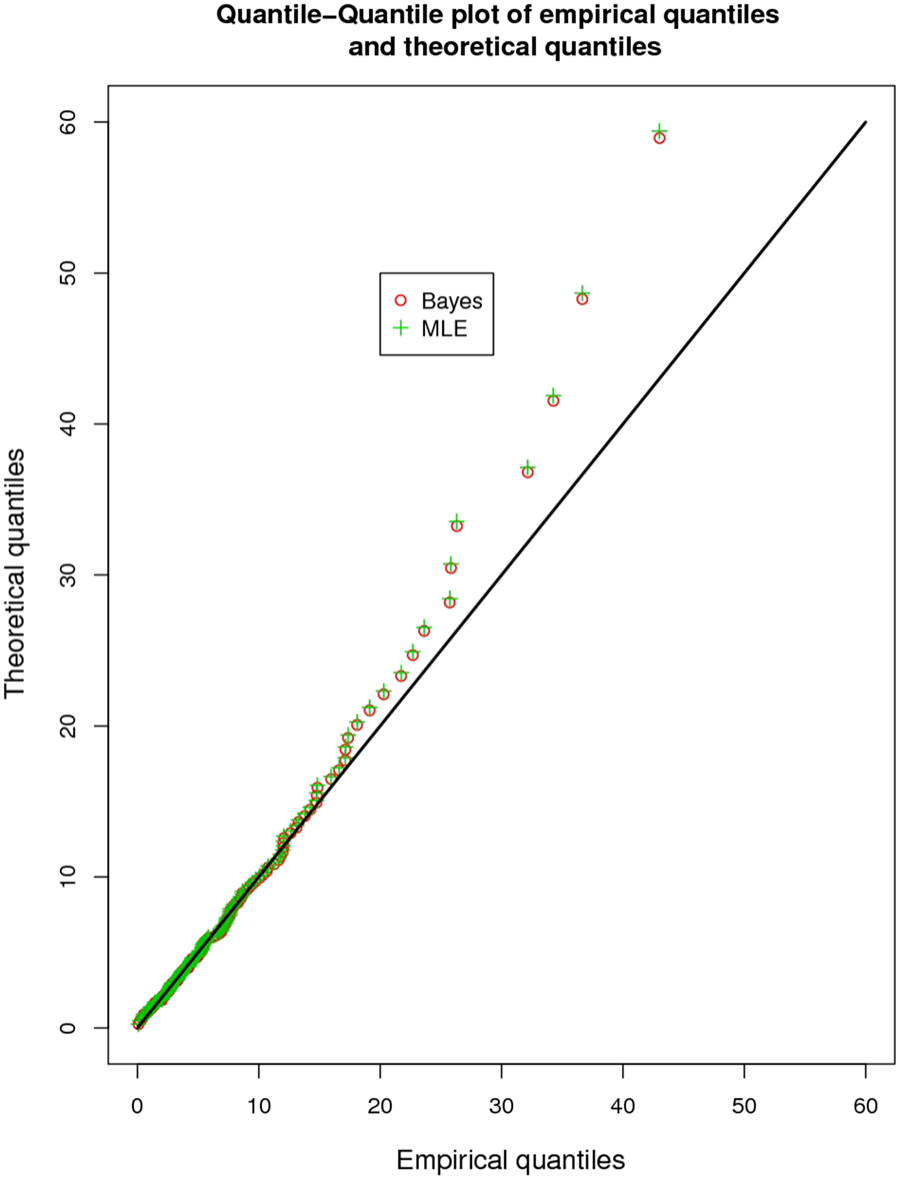
Q–Q plot of the quantiles using MLEs and Bayesian estimates

**Fig. 12:**
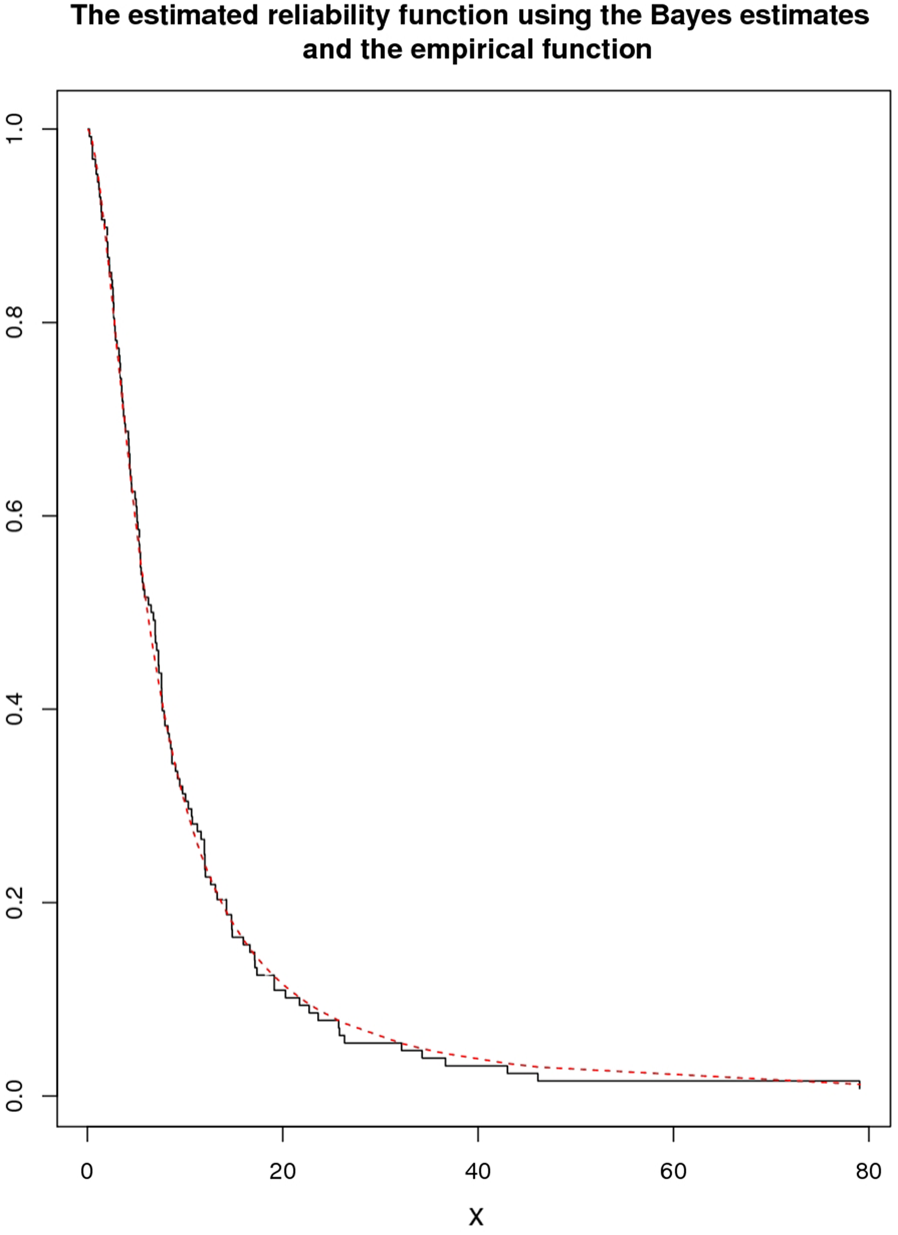
The estimated survival function using Bayes estimate and the empirical reliability function

Three graphs have been plotted Figs. 10, 11 and 12 for comparison of MLEs with Bayesian Estimates. Figure 10 represents the density functions of LL model based on MLEs and Bayes estimates, from uniform priors through the use of samples obtained by MCMC technique. It is witnessed that both density functions coincide.

Quantile–quantile (Q–Q) plot of empirical versus theoretical quantiles computed using MLEs and Bayes estimates is displayed in Fig. 11. Here also it is witnessed that the green circles depicting MLEs coincide with the red circles depicting Bayes estimation.

Estimated reliability function is displayed in Fig. 12 using Bayesian estimates calculated from uniform priors along with empirical reliability function.

Keeping in view the foregoing visual representations from Figs. 10, 11 and 12 using MLEs and the Bayes estimates based on uniform priors to a great extent coincide and suggests a good fit for the proposed LL model.

## Conclusion

Present research discussed the LL model with two parameters; MLEs and Bayesian estimates are obtained from a real life sample using the Markov Chain Monte Carlo (MCMC) technique using OpenBUGS software. Bayesian analysis under different set of priors has been carried out and convergence pattern was studied using different diagnostics procedures. Numerical summary based on MCMC samples from posterior distribution of LL model has been worked out based on non-informative priors. Visual review for different set of priors including box plot, kernel density estimation in comparison with MLEs has been attempted. It is witnessed that the LL model whether used with MLEs or with Bayesian Estimates fits the data well. It has been found that the proposed methodology is suitable for empirical modeling under uniform sets of priors. Although the simulation study is not conducted in the present work. But, the consistency, basic study and comparisons of present estimation and improved parameters estimation by Reath (2016) will be conducted in future work.
